# Construction of Superhydrophobic Coating on Iron Surface with Enhanced Anti-Corrosion, Anti-Adhesive and Anti-Bacterial Properties

**DOI:** 10.3390/ma15238634

**Published:** 2022-12-03

**Authors:** Wuyifan Zhou, Feng Yang, Ling Yuan, Yangmin Diao, Ou Jiang, Yuan Pu, Yong Zhang, Yong Zhao, Dan Wang

**Affiliations:** 1 Superconductivity and New Energy R&D Center, Southwest Jiaotong University, Chengdu 610031, China; 2 Key Laboratory of Advanced Technology of Materials (Ministry of Education of China), Key Laboratory of Magnetic Suspension Technology and Maglev Vehicle (Ministry of Education of China), School of Materials Science and Engineering, Southwest Jiaotong University, Chengdu 610031, China; 3 Oncology Department, The Second People’s Hospital of Neijiang, Neijiang 641000, China; 4 State Key Laboratory of Organic–Inorganic Composites, Beijing University of Chemical Technology, Beijing 100029, China

**Keywords:** iron surface treatment, superhydrophobic coating, anti-corrosion, anti-adhesive, anti-bacterial

## Abstract

Superhydrophobic coatings on iron surface have a wide application potential in medical instruments, chemical industrial equipment, and house construction. In this work, we developed a multi-functional superhydrophobic coating on iron surface with a high air/water contact angle of 162.3° and a low sliding angle of 2.4°. The construction of superhydrophobic coating involves physical friction processing to fabricate micropatterns and structures, followed by annealing treatment and surface chemical modification with 1H,1H,2H,2H-tridecafluoro-n-octyltrimethoxysilane. The obtained organic–inorganic composite material exhibited considerable optimization potential to anti-condensation performance. The low surface energy of the superhydrophobic coating also leads to poor adhesion of water, dust, and blood platelets, which is beneficial for applications in medical devices. The electrochemical and impedance test results demonstrated that the superhydrophobic surface provided effective corrosion protection for the iron substrate, with an 84.63% increase in corrosion protection efficiency. The experimental results showed that the anti-bacterial ratios reached 90% for *E. coli* and 85% for *S. epidermidis*, while the anti-bacterial ratios of ordinary iron were only 8% for *E. coli* and 15% for *S. epidermidis*, respectively.

## 1. Introduction

Superhydrophobic surfaces inspired by the non-wetting properties of lotus leaves in nature have been widely used in many fields such as underwater drag reduction [1], oil–water separation [2,3,4], self-cleaning [5,6], bio-/chemical assays [7,8,9], and civil applications [10,11,12] due to their unique characteristics of water resistance, anti-adhesion and anti-corrosion properties, which have wide application prospects and high research value. In particular, the strategy of constructing superhydrophobic surfaces or coatings on metal surfaces [13,14] to achieve multiple functions with enhanced anti-corrosion, anti-adhesive and anti-bacterial properties is of great significance to solve the problems of poor corrosion resistance and microbial adhesion infection on the surfaces of medical devices and equipment [15,16]. In order to minimize the risk of bacterial colonization, researchers have also been working on the design and development of functionalized surfaces to achieve long-lasting and stable antimicrobial performance [17,18,19,20]. However, broad application of the materials innovation-driven technologies will not be realized if there is no low-cost and large-scale fabrication capability for the superhydrophobic materials [21,22,23,24]. Most of the currently developed methods for construction of superhydrophobic coating are still limited by the complicated process and valuable equipment [25]. Iron and steel iron have been the most widely used materials for building the world’s infrastructure and industries, as well as the most common metals for surgical instruments, chemical and hazardous waste receptacles, prostheses, crutches, and metal plates [26,27,28]. Therefore, fabricating superhydrophobic surfaces on iron has a vital significance.

In this work, we report a simple and effective method for constructing superhydrophobic iron (SHI) with enhanced anti-corrosion, anti-adhesive and anti-bacterial properties. The iron-based superhydrophobic surfaces were prepared by physical friction processing to fabricate micropatterns and structures, followed by annealing treatment and surface chemical modification with 1H,1H,2H,2H-tridecafluoro-n-octyltrimethoxysilane to realize low surface energy. The air/water contact angle (CA), microstructure characterization, diffraction of X-rays and spectral analysis were employed to evaluate grafting effects and superhydrophobic performance. The anti-condensation, anti-platelet adhesion, electrochemical corrosion tests and anti-bacterial properties of the prepared samples were systematically classified.

## 2. Materials and Methods

### 2.1. Materials and Characterization

Iron (purity ≥ 99.5%) was purchased from Sigma-Aldrich. The 1H,1H,2H,2H-tridecafluoro-n-octyltrimethoxysilane (FAS-13, C_11_H_13_F_13_O_3_Si, 97%) was purchased from Aladdin Reagent (Shanghai) Co. Ltd. (Shanghai, China). Stearic acid (STA, C_18_H_36_O_2_) was purchased from Tianjin Chemical Industry Co. (Tianjin, China). All other reagents were analytically pure and were used as received without purification unless it is specifically requested. Two representative strains, namely Staphylococcus epidermidis (*S. epidermidis*, ATCC 12228) and Escherichia coli (*E. coli*, ATCC 25922), were obtained from Shanghai Luwei Technology Co. The surface wettability was measured by the air/water CA of the contact angle system DSA100 (Krüss, Hamburg, Germany) using a 5 μL drop of water as an indicator. The microstructure and morphologies of the samples were studied by using a field emission scanning electron microscope (SEM, Hitachi S-4800, Tokyo, Japan). The crystal structures of the samples were analyzed by X-ray diffraction (XRD, MAC Science MXP-3VA, Tokyo, Japan) with a Cu Kα radiation source and the tests were performed in the range of 20~80° with a scan rate of 2θ = 10° min^−1^. The Fourier transform infrared spectroscopy (FTIR, Thermo Scientific Nicolet iS50, Waltham, MA, USA) was used to analyze the chemical grafting of the iron-based samples. The electrochemical corrosion tests were performed on an electrochemical workstation (IM6, Zahner, Kronach, Germany) in a 3.5% NaCl solution at 25 °C. Optical microscope photographs were taken under a digital biological microscope (XSP-9CE, Shanghai Changfang Optical Instrument Co., Ltd., Shanghai, China).

### 2.2. Preparation of Superhydrophobic Coating on Iron Surface

Typically, iron substrates with an average size of 10 × 10 × 1 mm were firstly cleaned completely with acetone and ethanol sequentially in an ultrasonic machine for 15 min to remove impurities, and were then dried in an N_2_ stream. Carved iron was obtained by scribing the substrate surface at 0.5 cm/s in several directions horizontally, vertically and obliquely using a scriber. Subsequently, the obtained iron samples were placed in a tube furnace and annealed in air at various temperature (400, 450, and 500 °C) for a certain time (2, 3, 4, and 5 h) to obtain samples of structured iron. Then, the samples of structured iron were immersed in 0.5 wt% FAS-13 ethanol solution for 12 h, followed by drying under nitrogen flow to superhydrophobic iron samples marked as SHI (FAS-13). For comparison, the superhydrophobic coating on iron modified with STA were also fabricated following previous literature [29,30]. The samples of structured iron were submerged in 0.02 mol/L STA ethanol solution for 12 h and were then dried under nitrogen flow to obtain superhydrophobic iron samples marked as SHI (STA).

### 2.3. Anti-Condensation Performance

High air humidity was simulated by spraying atomized water droplets at a uniform rate 3 cm above the sample, and the condensation of droplets on the sample surface was recorded and weighed at regular intervals. The condensation rate of each group of samples was compared to assess the performance of the samples against condensation. The condensation rate of each sample was calculated according to Equation (1).

(1) $$f=\frac{W_{2}-W_{1}}{W_{1}}\times 100\%$$

where *f* is the condensation rate of the test iron sample, *W*_1_ is the initial mass of the test sample, and *W*_2_ is the mass of the test iron sample after condensation.

### 2.4. Anti-Platelet Adhesion Test

Briefly, 5 mL of fresh whole blood from sterile rabbits was centrifuged in a high-speed centrifuge at 1500 r/min for 15 min. Blood was centrifuged and layered, and the upper layer of yellow transparent platelet-rich plasma (PRP) was aspirated. Then, 200 μL of PRP was pipetted dropwise onto the surface of the three sets of samples. The samples were then incubated in water bath of 37 °C for 50 min. After being gently rinsed with saline, the samples were fixed with glutaraldehyde for another 12 h. After dehydration in gradients of 50%, 75%, 90%, and 100%, the adhesion of platelets on samples was investigated under a digital biological microscope.

### 2.5. Electrochemical Corrosion Test

The electrochemical corrosion behaviors of the samples, including the dynamic potential polarization (PDP) and electrochemical impedance spectroscopy (EIS), were investigated using an electrochemical workstation (IM6, Zahner, Germany) with a three-electrode cell system consisting of a reference electrode (saturated glycerol electrode), a counter electrode (platinum) and a working electrode (test sample). The back side of the test sample was sandpapered and connected with copper wire. The sample was then sealed with silicone rubber to expose the test surface with an area of 1 cm^2^. NaCl solution (3.5%) was used as the electrolyte. Before conducting the test, the sample was immersed in the NaCl solution for 30 min to stabilize the surface and finally achieve a relatively stable open circuit potential. The PDP tests were performed at an applied potential range from −500 mV to +500 mV relative to the open circuit potential with a scan rate of 1 mV/s. The self-corrosion potential (*E_corr_*) and self-corrosion current density (*I_corr_*) were calculated from the polarization curves using the Tafel linear extrapolation method, respectively. The corrosion protection efficiency (ƞ) was obtained from Equation (2):

(2) $$\eta =\left(I{{}^{\circ}}_{corr}-I_{corr}\right)/I{{}^{\circ}}_{corr}\times 100\%$$

where *I_corr_* is the free corrosion current density of the pure iron sample and *I_corr_* is the free corrosion current density of the SHI sample.

The EIS spectra were recorded in the frequency range of 200 kHz to 0.01 Hz with a sinusoidal perturbation signal of 10 mV (peek to peek). The results were fitted and analyzed with ZSimpWin software (V 3.60). For electrochemical corrosion experiments, each test was repeated 4 times and the average value was calculated.

### 2.6. Anti-Bacterial Test

Three groups of samples—ordinary iron (Iron), SHI (STA), and SHI (FAS-13)—were sterilized by UV light and placed in sterile Petri dishes. The *E. coli* and *S. epidermidis* were precultured in agar solid medium, positioned in an incubator at 37 °C for 24 h and passaged twice to obtain monoclonal bacteria. Fresh bacterial colonies (1–3 rings) were picked and lysed in a solution containing 0.2% liquid medium and 99.8% saline. The bacterial concentration was reconstituted to 5.0 × 10^5^ CFU mL^−1^ by sequential dilution in tenfold increments. Then, 200 μL of the appropriate concentration of bacterial solution was added to the sample surface and all samples were incubated at 37 °C for 24 h. At the end of incubation, the samples were gently removed and rinsed slowly with saline. Then, 30 μL of bacteria-containing saline was added to the surface of the solid medium for plate coating, which was continued upside down for 24 h. After that, the bacteria were photographed and counted, and the antimicrobial rate R was calculated according to the Equation (3).

(3) $$R=\left(N_{c}-N\right)/N_{c}\times 100\%$$

where *N_c_* and *N* are the number of colonies on the control and experimental group samples, respectively.

Each test was repeated 3 times and the average value was calculated.

## 3. Results and Discussion

### 3.1. Surface Morphology, Composition and Wetting Properties

**Figure 1:**
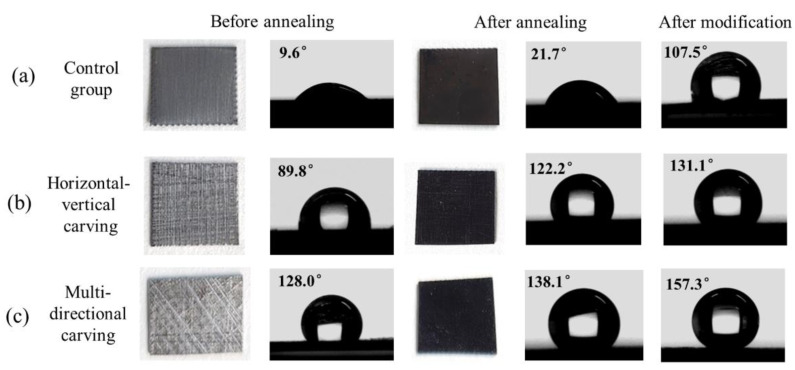
Digital photos and CA results of (**a**) ordinary iron, iron samples treated by (**b**) horizontal–vertical carving and (**c**) multi-directional carving under different treatment including annealing and surface modification.

To obtain robust superhydrophobic coating on iron surface, micron-level rough structures were firstly constructed on the iron surface by different scribing methods. The samples were then thermally oxidized to generate nanostructures on the micron structures to realize the construction of multi-level rough structures on the iron-based surface. Figure 1 shows the macroscopic structures on the surface of ordinary iron, iron samples treated by horizontal–vertical carving and multi-directional carving. The CA of the iron-based samples increased from 9.6° to 89.8° and 128.0° after scribing in different ways. The photos of the samples after annealing treatment were also present. Due to the surface oxidation of iron, the samples appeared black in color. The annealing treatment could make the CA of samples improve to 21.7°, 122.2° and 138.1° for ordinary ion, iron samples treated by horizontal–vertical carving and multi-directional carving, respectively, which was attributed to the formation of iron oxide nanostructures during the annealing. Furthermore, after modification by low-surface-energy molecules of FAS-13, the CA values were further improved to 107°, 131.1° and 157.3°. These results demonstrate that construction of micropatterns and surface chemical modification have associated action in promoting the superhydrophobic surface on iron. In addition, we use two-liquid method to estimate the surface energy of the coatings following the method in previous literature [31]. Water and glycerol were used as the two liquids for calculations in our work. The dispersive and polar components of surface tension of water are 21.8 and 51.0 mN/m, respectively, whereas these values for glycerol are 37 and 26.4 mN/m, respectively. The water contact angle is 157.3°, while the values measured for glycerol is 155.0°, respectively. The above values were utilized and surface energy of the SHI was calculated as 0.51 mN/m.

**Figure 2:**
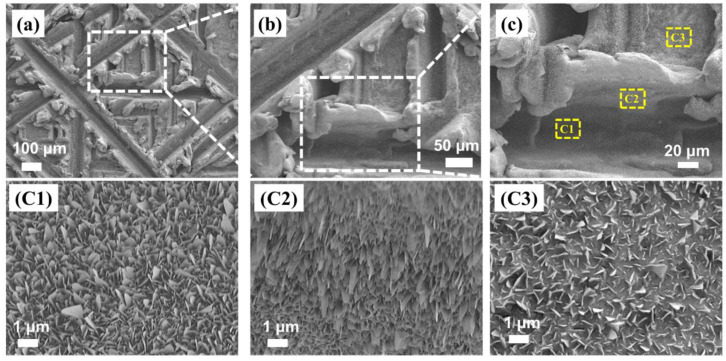
(**a**) SEM images of FAS-13-modified superhydrophobic iron; (**b**) enlarged image in corresponding box of (**a**); (**c**) enlarged image in corresponding box of (**b**); (**C1**–**C3**) enlarged images in corresponding boxes of (**c**).

The samples of FAS-13-modified superhydrophobic iron were then investigated b SEM. Figure 2 shows the rough micro/nano structure of the sample in different orientations. In the inner part of the trench of C1 and the side of C2 in Figure 2c, there were extremely dense nanosheets approximately 1~3 nm thick and 10~500 nm wide. In the area C3 in Figure 2c, the nanosheets were approximately 10~30 nm thick and 50~500 nm wide, which might have lower densities with better mechanical strength. The morphological investigation of the SHI samples showed that the rough structure with graded micron structure and nanostructure was established by scratching and annealing sequentially. This surface morphology with a rough hierarchical micro- and nanostructure helps the sample to trap a stable air layer on the surface, which can lead to a higher surface contact angle and prevent water and other liquids from wetting the surface [29].

**Figure 3:**
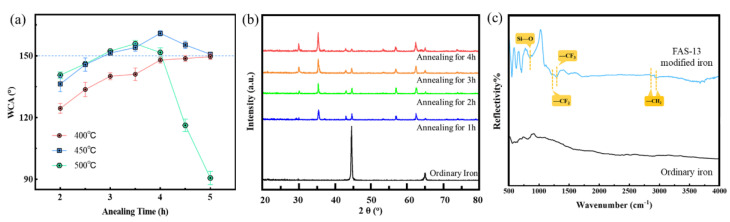
(**a**) Wettability contact angle (WCA) profiles of various samples annealed at 400, 450 and 500 °C for different times (2, 3, 4, and 5 h, respectively); (**b**) XRD patterns of iron samples treated at 450°C for different times (0, 1, 2, 3, and 4 h); (**c**) FTIR spectra of ordinary iron and superhydrophobic iron modified with FAS-13.

The effect of annealing conditions on the wettability of the SHI samples was also investigated. As shown in Figure 3a, when the samples were annealed at 400 °C for 4~5 h, their CA could be significantly increased to approximately 150°. When the samples were annealed at 450 °C for 3~5 h, the CA of the samples was higher than 150°. For the samples under annealing treatment for 4 h, the static water CA of the sample reached 162.3° along with SA of 2.4°. When the annealing temperature was further increased to 500 °C, the surface structure was unstable and the oxide layer peeled off from the surface of the 4~5 h annealed samples. Therefore, the superhydrophobic samples were prepared by annealing at 450 °C for 4 h, which was the optimized annealing condition for wettability. The crystal structures of the samples were investigated by XRD analysis (Figure 3b). Two distinct peaks at 44.67° and 64.99° in the curve of the pure iron sample were diffracted from the crystal planes of the pure iron. After annealing treatment for 60 min, the sample curves show diffraction peaks of Fe_2_O_3_ and Fe_3_O_4_, indicating that the iron on the surface was oxidized by oxygen in air [29,32]. The peak intensity of iron oxides increased with the extension of annealing time. The FTIR spectra of iron and FAS-13-modified SHI were present in Figure 3c. For the SHI (FAS-13) samples, the Si—O symmetric stretching vibration absorption peak at 850 cm^−1^ and the stretching vibration of the —CF_2_ and —CF_3_ groups at 1220 and 1293 cm^−1^ were observed [33], along with antisymmetric stretching vibration and symmetric stretching vibration absorption peaks of —CH_2_ at 2859 and 2942 cm^−1^, respectively [34]. These results indicated that the low-surface-energy organic materials of FAS-13 were successfully grafted.

### 3.2. Anti-Condensation Performance

**Figure 4:**
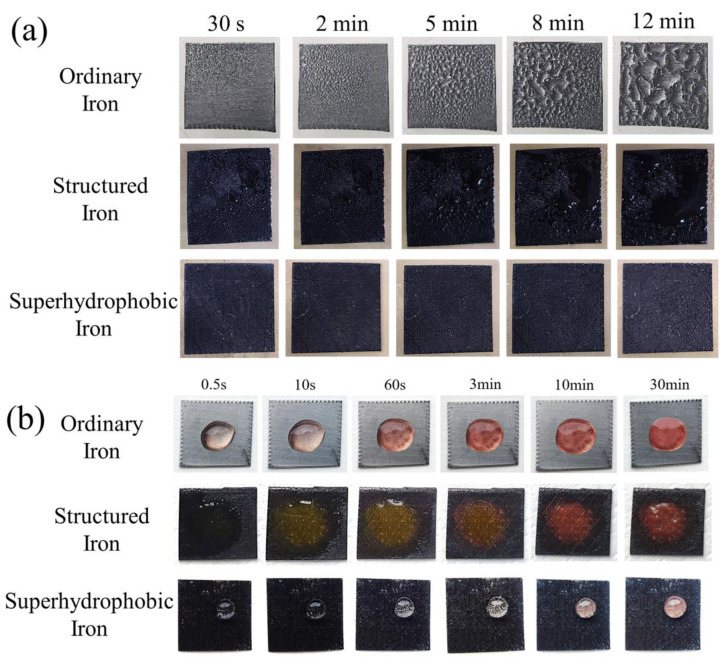
(**a**) Photos of three samples—ordinary iron, structured iron and SHI—for anti-condensation tests as time prolonged to 12 min. (**b**) Photos of droplets of hydrochloric acid (pH = 1) on the surface of the ordinary iron, structured iron and SHI as time prolonged to 30 min.

In practical applications, when the relative humidity of the air is high, metal surfaces usually condense water molecules to form water films and water droplets. This provides conditions for the migration of harmful ions and makes it easier for electrochemical corrosion to occur. Therefore, improvement of the anti-condensation performance of iron can slow down the metal corrosion and improve the durability of the structure to a certain extent. Figure 4a shows the photos of three samples—ordinary iron, structured iron and SHI—for anti-condensation tests. On the surface of ordinary iron, there were a few small droplets condensing locally from 30 s. The surface of ordinary iron was almost fully covered by water film after condensation test for 2 min, and the condensed droplets on the surface had a significant increase as time prolonged to 12 min. According to quantitative analysis, 25.0 mg of droplets had condensed on the surface of the ordinary sample, with a condensation rate calculated as 7.8%. For the structured iron samples, a little ultrafine droplets of water condensed on the surface within 30 s. However, gathering of water on the surface were not statistically significant as time prolonged to 12 min. The condensation rate of structured iron samples was calculated as 4.5%. The liquid beads exhibited a small contact angle after condensing and the droplets gradually infiltrated on the surface. As expected, the surface of the SHI samples was almost free of liquid droplet condensation for the first 5 min. After testing for 8 min, small liquid droplets were observed on the surface of SHI; and as time prolonged to 12 min, the condensation rate of SHI was calculated as 1.1%. In addition, the anti-acid corrosion characteristics of ordinary iron, structured iron and SHI were investigated. Droplets of hydrochloric acid (pH = 1) were placed on the surface of the samples and the photos were taken once in a while for up to 30 min. As the results show in Figure 4b, both the ordinary iron and structured iron were easily corroded, resulting in a color change in the droplets from colorless to red. The coating of FAS-13 on the SHI could effectively shield the metallic iron and iron oxides from acid corrosion. The quantitative measurements indicated the mass loss per area of the samples was 134, 54, and 7 mg/cm^2^ for the ordinary iron, structured iron and SHI.

### 3.3. Self-Cleaning Performance and Anti-Platelet Adhesion Properties

**Figure 5:**
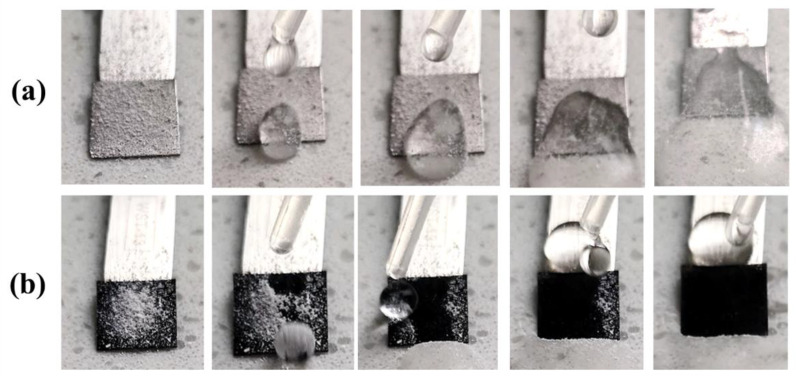
Self-cleaning test images for (**a**) ordinary iron and (**b**) SHI (FAS-13) samples.

To characterize the self-cleaning performance of the sample, the samples were firstly tilted at 5° and covered with dust on the surface. Dynamic droplets of water were released onto the sample. As shown in the photos recorded in Figure 5, the droplets of water fell on to the surface of ordinary iron covered with dust were easily trapped. A small portion of the dust could be separated from the surface of the water droplets; however, most of them adhered to the surface of iron, which might cause further contamination and negative effects on the use of the materials. A noticeable difference was observed on the surface of SHI. The powder rolled down with the water droplet and was completely removed with the increased volume of water drops. These results demonstrated that the obtained samples of SHI exhibited a good self-cleaning property. The self-cleaning property of superhydrophobic surface can reduce the adsorption of pollutants and microorganisms in the environment, and even if it is adsorbed, the surface can be cleaned by natural precipitation and other physical effects to achieve the purpose of anti-fouling, anti-mildew, anti-biological adhesion and self-cleaning.

**Figure 6:**
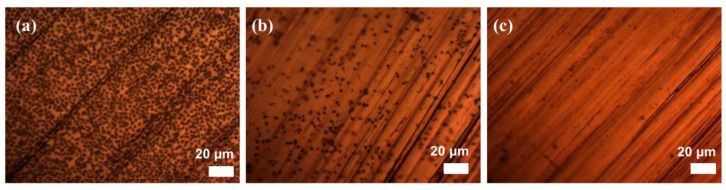
Platelet adhesion images acquired under different amplification times observed under optical microscopy for (**a**) ordinary iron, (**b**) structured iron and (**c**) superhydrophobic iron.

The adsorption and activation of platelets on the material surface is a major cause of blood clotting and thrombosis, which largely affects the use of implants and other blood-contact devices, where altering the surface wettability of the material becomes an important means of achieving anti-coagulation and anti-thrombosis. Figure 6 shows the platelet adhesion images acquired under different amplification times observed under optical microscopy for three groups of samples—ordinary iron, structured iron and superhydrophobic iron. The untreated normal iron surface had a large number of platelets adhering, while the structured iron surface had a relatively small number, less than one-tenth of the normal iron surface adhesion. In contrast, the superhydrophobic surface had only a small number of platelets adhering due to the air layer. The results of platelet adhesion experiments showed that the rough structure with low surface energy could significantly improved the anti-platelet adhesion performance of the materials, and inhibited platelet activation and pseudopod formation, avoiding the formation of thrombus and blood clotting, which has potential research value for the construction of superhydrophobic surfaces on instrument surfaces in contact with blood.

### 3.4. Electrochemical Corrosion Test

**Figure 7:**
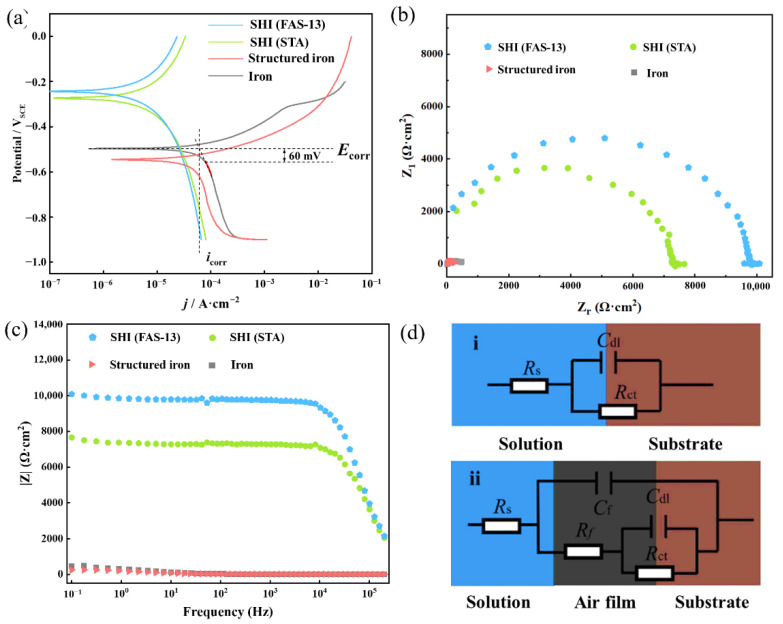
(**a**) PDP curves (**b**) Nyquist plot and (**c**) Bode plot measurements of ordinary iron, structured iron, SHI (STA) and SHI (FAS-13) in 3.5% NaCl solution. (**d**) Equivalent circuit EIS results for (**i**) ordinary iron, (**ii**) structured iron, SHI (STA) and SHI (FAS-13) samples.

Furthermore, the electrochemical corrosion tests were carried out to determine the change in the corrosion property. In these experiments, the STA-modified superhydrophobic coating on iron was also investigated for comparison. The kinetic potential polarization curves and EIS test results for the four groups of samples—ordinary iron, structured iron, SHI (STA) and SHI (FAS-13)—are presented in Figure 7. Briefly, the self-corrosion potential *E_corr_* and self-corrosion current density *I_corr_* reflect the thermodynamic state of the sample surface, which can further reflect the ease of corrosion occurring in the samples. The more positive the self-corrosion potential *E*_corr_ and the lower the self-corrosion current density *I_corr_* of the sample, the less it has the tendency to be corroded, the lower the degree and chance of being corroded, and the smaller the corrosion rate in the test solution [35]. It can be seen from Figure 7a that after annealing of iron, the self-corrosion potential shifted downward from −0.50 V (iron) to −0.55 V (structured iron), while the self-corrosion potential of the samples after low-surface-energy modification had a large upward shift, positively increasing to −0.27 V and −0.23 V for SHI (STA) and SHI (FAS-13), respectively. The self-corrosion current densities *I_corr_* of the two superhydrophobic iron samples of SHI (STA) and SHI (FAS-13) were 0.12 and 0.083 μA cm^−2^, respectively, which were much lower than those of 0.54 μA cm^−2^ for ordinary iron and 1.44 μA cm^−2^ for structured iron. Accordingly, the corrosion protection efficiency of the superhydrophobic iron samples was improved by 77.78% and 84.63%, respectively (Table 1). The resultant rough structure of the surface with low surface energy can improve the thermodynamic stability of the iron surface and slow down its corrosion tendency in 3.5 wt% NaCl solution. Nyquist plot (Figure 7b) and bode plots (Figure 7c) of four groups of samples measured by EIS method in 3.5 wt% NaCl solution demonstrated the resultant rough structure of the surface with low surface energy can improve the thermodynamic stability of the iron surface and slow down its corrosion tendency. The Nyquist plot shows that the diameter of the semicircle in the Nyquist loop of the samples modified with low surface energy was larger than that of the unmodified samples. The sample modified with FAS-13 exhibited the largest semicircle diameter, which confirms it had the best corrosion resistance among all samples. The total impedance of the low-surface-energy-modified samples was found to be significantly higher than those of unmodified samples from the Porter plot (Figure 7c). The equivalent circuits fitting the impedance results are shown in Figure 7d. In these circuits, *R*_s_ represents the solution resistance; *C*_dl_ and *R*_ct_ represent the double-layer capacitance and charge transfer resistance, respectively; C_f_ represents the capacitance of the air layer; and *R*_f_ represents the total resistance of the pore or coating. The equivalent circuit in Figure 7d(i) was used to fit the EIS data of pure iron, and equivalent circuit in Figure 7d(ii) was used to fit the EIS data of structured iron, SHI (STA) and SHI (FAS-13) samples. According to Table 2, the releasing of corrosion products of iron and structured iron resulted slightly different *R*s. Due to the large amount of air pockets trapped in the hierarchical micronanostructures between liquid and solid, the *R*_f_, *C*_dl_, and *R*_ct_ of SHI (STA) and SHI (FAS-13) were significantly improved, indicating that the superhydrophobic surface significantly improved the corrosion resistance of the substrate [30]. Since the *R*_f_, *C*_dl_, and *R*_ct_ of SHI (FAS-13) were larger than those of SHI (STA), The SHI (FAS-13) exhibited better performance on electrochemical corrosion resistance.

### 3.5. Anti-Bacterial Properties

**Figure 8:**
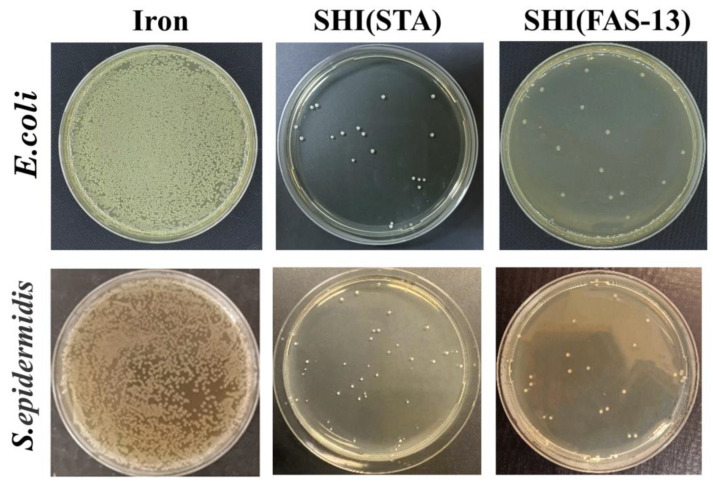
*E. coli* and *S. epidermidis* on ordinary iron (Iron), and superhydrophobic iron samples of SHI (STA) and SHI (FAS-13).

The results of anti-bacterial tests by the flat plate method are shown in Figure 8. The proliferation of *E. coli* and *S. epidermidis* were recorded and analyzed. On the surface of the ordinary iron, the *E. coli* and *S. epidermidis* grew vigorously and covered a large area of the medium. According to the statistical results of the anti-bacterial rate obtained by ImageJ software (V 1.8.0.345) statistics and calculation, the anti-bacterial rate of the ordinary iron samples was only 8.15 ± 0.6% and 15 ± 2.55% for *E. coli* and *S. epidermidis*, respectively. However, the two groups of superhydrophobic iron samples showed significantly enhanced anti-bacterial performance with a significant reduction in the number of colonies. Both SHI (STA) and SHI (FAS-13) exhibited similar anti-bacterial rates of over 90% for *E. coli* while the anti-bacterial rates for *S. epidermidis* were approximately 85~87%. The results demonstrated that low-surface-energy coatings on iron surfaces have significant effects on the anti-bacterial performance of the samples. The anti-bacterial rates were related to the types of bacteria and less related to the surface modified materials. Our preliminary study illustrated a promising technology for construction of superhydrophobic coating on iron surface with desirable properties for advanced applications of iron-based devices such as chemical and hazardous waste receptacles, and prostheses.

## 4. Conclusions

In this work, a simple and effective method was demonstrated for construction of superhydrophobic coating on iron surface with enhanced anti-corrosion, anti-adhesive and anti-bacterial properties. The superhydrophobic surface was prepared on the iron substrate surface by a combination of simple mechanical processing, heat treatment, and low-surface-energy modification, without the need for special use of expensive equipment. Meanwhile, the obtained samples exhibited a high air/water contact angle up to 162.3° and a low sliding angle. The superhydrophobic surface enabled the iron to be highly water repellent, with significant water repellency and better anti-condensation performance than the ordinary iron samples. The superhydrophobic iron samples also exhibited good mechanical stability and corrosion resistance. The low surface energy of the superhydrophobic coating also leads to poor adhesion of water, dust, and blood platelets. The low adhesion property of the surface brings better anti-bacterial quality. The strategy is applicable for surface engineering of iron or carbon steel with iron over 90%. For substrates with high hardness and high-wear-resistance coatings, it is difficult to construct microstructures by physical friction processing. It should be out that several kinds of techniques have been developed for the construction of superhydrophobic coating, including but not limited to the surface painting of stearic acid, fluorine-containing acrylic oligomers, and polyhedral oligomeric silsesquioxane. The synergism of nano/microhierarchical rough structures and chemical modification also plays an important role. It is not difficult to compare our samples with all the other types of superhydrophobic coatings obtained by stearic acid, fluorine-containing acrylic oligomers, etc. Actually, the overall performance of coatings based on the same kind of chemical modification reactants is usually not the same. The performance of FAS-13-modified iron can be further improved by optimizing experimental conditions (e.g., the treatments of friction, the concentrations of FAS-13), and the performance comparisons of our technique with others should be an interesting topic for follow-up studies. Nevertheless, the preliminary studies in this work illustrated a promising technology for construction of superhydrophobic coating on iron surface with desirable properties for advanced devices.

## Figures and Tables

**Table 1:** Self-corrosion potential, self-corrosion current and calculated corrosion rate of different treated samples.

Sample	*E*_corr_ (V_SCE_)	*I*_corr_ (μA·cm^−2^)	η (%)
Iron	−0.50	0.54	—
Structured iron	−0.55	1.44	−166.67
SHI (STA)	−0.27	0.12	77.78
SHI (FAS-13)	−0.23	0.083	84.63

**Table 2:** Fitting results of electrochemical impedance mapping.

Sample	*R*_s_ (Ω·cm^2^)	*C*_f_ (μF·cm^−2^)	*R*_f_ (Ω·cm^2^)	*C*_dl_ (μF·cm^−2^)	*R*_ct_ (Ω·cm^2^)
Iron	14.68	—	—	180.3	250.4
Structured iron	14.70	33.45	71.94	92.12	453.5
SHI (STA)	17.94	2.94 × 10^−4^	231.3	591.4	7231
SHI (FAS-13)	17.60	3.07 × 10^−4^	246.8	805.3	9766

## Data Availability

Not applicable.
